# Knockdown of ubiquitin-specific peptidase 39 inhibited the growth of osteosarcoma cells and induced apoptosis in vitro

**DOI:** 10.1186/s40659-017-0121-z

**Published:** 2017-04-12

**Authors:** Zhihua Gan, Kun Han, Shuchen Lin, Haiyan Hu, Zan Shen, Daliu Min

**Affiliations:** Department of Medical Oncology, The Affiliated 6th People’s Hospital of Shanghai Jiaotong University, Shanghai, 200223 China

**Keywords:** Cell cycle, Migration, Osteosarcoma, Proliferation, USP39

## Abstract

**Background:**

Ubiquitin specific peptidase 39 (USP39), an essential factor in the assembly of the mature spliceosome complex, has an aberrant expression in several cancer. However, its function and the corresponding mechanism on human osteosarcoma has not been fully explored yet.

**Methods:**

The mRNA and DNA copies of USP39 were increased in osteosarcoma cancer tissues compared with the one in human normal tissues according to datasets from the publicly available Oncomine database. A further western blot analysis also demonstrated an aberrant endogenous expression of USP39 in three different osteosarcoma cells. Then lentivirus-mediated short hairpin RNA (shRNA) was designed to silence USP39 in human osteosarcoma cell line U2OS, which is used to test the impact of USP39-silencing on cellular proliferation, colony formation, cell cycle distribution and apoptosis.

**Results:**

Knockdown of USP39 expression in U2OS cell significantly decreased cell proliferation, impaired colony formation ability. A further analysis indicated suppression of USP39 arrested cell cycle progression at G2/M phase via p21 dependent way. In addition, the results of Annexin V/7-AAD staining suggested the knockdown of USP39 could promote U2OS cell apoptosis through PARP cleavage.

**Conclusions:**

These results uncover the critical role of USP39 in regulating cancer cell mitosis and indicate USP39 is critical for osteosarcoma tumorigenesis.

## Background

Osteosarcoma derives from primitive bone-forming mesenchymal cells and is the most common primary malignancy of bone in children and adolescents [1–3]. It is the eighth common form of childhood cancer and its incidence rate in the United States patients under 20 years old is estimated at 5.0 (4.6–5.6) per million per year [4]. Five-year survival rates for children and adolescents were improved to around 70% when employed chemotherapy and definitive surgical resection in the early 1980s [5]. However, although more and more novel assessment methods are applied to diagnose osteosarcoma [6], improvement in osteosarcoma survival during last decade has been postponed, patients with osteosarcoma are in urgent needs for new treatment strategies.

Ubiquitin specific peptidase 39 (USP39) encodes a conserved 65 kDa SR-related protein in humans, which is crucial for eukaryotic gene expression and any defective splicing can be detrimental [7]. It is proved to be involved in assembly of the RNA spliceosome [8, 9]. The spliceosome consists of five small nuclear ribonucleoproteins (snRNPs). Human 65 kDa SR-related protein is essential for the recruitment of the tri-snRNP-specific protein [10, 11]. Furthermore, USP39 was identified as a new factor required to maintain the spindle checkpoint and support successful cytokinesis [12], and classified as a deubiquitinating enzyme [13–15]. Recently, scientists reported aberrant USP39 expression could inhibit breast cancer cell growth in vitro [16], however, little is known about how USP39 functions in human osteosarcoma and whether it can be used as an potential therapeutic target. In this present study, we employed a lentivirus-mediated RNA interference technique, a powerful tool to carry out loss-of-function assays in the investigation of cancer gene therapy [17, 18], to achieve highly stable silencing of USP39 in osteosarcoma cell line U2OS. To our knowledge, this is the first presentation providing evidence that the expression of USP39 is increased in osteosarcoma cancer tissues compared with the one in human normal according to datasets from the publicly available Oncomine database. Knockdown of endogenous USP39 expression could suppress the oncogenic properties of osteosarcoma cells and induce cell cycle arrest at G2/M phase, promote apoptosis through PARP cleavage. Our findings may shed light on the preliminary mechanisms underlying the tumorigenesis progression of osteosarcoma.

## Methods

### Cell culture

Human embryonic kidney cell line 293T (HEK293T), human bone sarcoma cells line SW1353, human primary osteogenic sarcoma cells line Saos-2 and human osteosarcoma cell line U2OS were obtained from Shanghai Institute of Cell Biology, the Chinese Academy of Sciences, and cultivated in DMEM medium (Hyclone, Logan, UT, USA), supplemented with 10% FBS (Biowest, Kansas City, MO, USA) at 37 °C in a humidified atmosphere of 5% CO_2_.

### Analysis of Oncomine data

To determine the expression pattern of USP39 in osteosarcoma, three datasets, including TGCA Sarcoma, Barretina Sarcoma and Detwiller Sarcoma in Oncomine database (https://www.oncomine.org) were used. The gene expression of USP39 was compared between osteosarcoma tissues with normal human bone according to the standard procedures [19].

### Construction of recombinant lentivirus targeting USP39

shRNA sequences of USP39 gene (NM_001256725.1) were from Sigma (http://www.sigmaaldrich.com/catalog/genes/USP39?lang=zh&region=CN#shRNA products) and the sequences were as follows:

shRNA-S1:

5′-CCTTCCAGACAACTATGAGATCTCGAGATCTCATAGTTGTCTGGAAGGTTTTT-3′.

shRNA-S2

5′-GATTTGGAAGAGGCGAGATAACTCGAGTTATCTCGCCTCTTCCAAATCTTTTT-3′.

negative control siRNA

5′-CAACAAGATGAAGAGCACCAACTCGAGTTGGTGCTCTTCATCTTGTTGTTTTTT-3′.

The shRNAs oligos were synthesized, annealed, and ligated into the BamHI/EcoRI-linearized shRNA vector pGreenPuro-L (SBI, USA), which contains a green fluorescent protein (GFP) as a reporter gene. This recombined pGreenPuro-L vector was transfected into the HEK293T cells together with lentiviral packaging mix (Sigma, USA) using Lipofectamine 2000 (Invitrogen, Carlsbad, CA, USA) according to the manufacturer’s instructions. For lentivirus transduction, U2OS cells were cultivated in six-well plates and inoculated with recombinant lentiviruses shCon, shUSP39-S1 and shUSP39-S2) at an MOI of 20. The infection efficiency was determined by counting the numbers of GFP-expressing cells under fluorescence microscope in 96 h.

### RNA extraction and real-time PCR

RNA extraction was prepared using trizol reagent (Gibco RL, Grand Island, NY, USA) according to the manufacturer’s instruction. 1 μg of total RNA was used to synthesize the first strand of cDNA using SuperScript II RT 200 μ/ml (Invitrogen, Carlsbad, CA, USA). USP39 mRNA expression was evaluated by real-time PCR on BioRad Connet Real-Time PCR platform (BioRad, Hercules, CA, USA) with SYBR Green PCR core reagents. The PCR system contains 2 × SYBR premix ex taq 10 µl, plus forward and reverse primers (2.5 µM) 0.8 µl, cDNA template 5 µl, and ddH2O 4.2 µl. β-Actin was applied as the internal reference. The following primers were synthesized and applied, USP39: 5′-GCCAGCAGAAGAAAAAGAGC-3′ as forward and 5′-GCCATTGAACTTAGCCAGGA-3′ as reverse; β-actin: 5′-GTGGACATCCGCAAAGAC-3′ as forward and 5′-AAAGGGTGTAACGCAACTA-3′ as reverse. The reaction procedure was initiated with denaturation at 95 °C for 1 min and followed by 40 repeated cycles (denaturation at 95 °C for 5 s and annealing extension at 60 °C for 20 s). The Ct-value for each sample was calculated with the ΔΔCt-method, and the results were expressed as 2^−ΔΔCT^ to analyze the fold change (tumor vs. normal): ΔΔCT = (CT target gene − CT actin) normal-(CT target gene − CT actin) tumor [20].

### Western blot analysis

Cells were washed twice with cool PBS and lysed in 2 × SDS sample buffer (10 mM EDTA, 4% SDS, 10% Glycine in 100 mM Tris–HCl, pH6.8). Equal amount of proteins (10 µg) were loaded and separated by electrophoresis (50 V, 3 h) on 10% SDS-PAGE gels and transferred to polyvinylidene difluoride (PVDF) membranes (Millipore, Bedford, MA, USA). The membranes were blocked and then probed with primary antibodies overnight, and then washed three times with TBST. Subsequently, the membranes were then incubated with horseradish peroxidase-conjugated secondary antibodies (1:5000, Santa Cruz) for 2 h at room temperature. After washing the blots were visualized by super ECL detection reagent (Applygen, Beijing, China). The primary antibodies were rabbit anti-USP39 (1: 1000, 23865-1-AP, Proteintech), rabbit anti-PARP (1:1000, #9542, Cell Signaling), rabbit anti-Caspase-3 (1:500, 25546-1-AP, Proteintech), rabbit anti-CDK1 (1:1000,19532-1, Proteintech), rabbit anti-cyclin A2 (1:1000,18202-1-AP, Proteintech), rabbit anti-cyclin B1 (1:1000, #21540, SAB), rabbit anti-p21 (1:1000, #2947, cell signaling), and mouse anti-GAPDH (1:500,000, HRP-60004, Proteintech).

### MTT viability assay

Methylthiazol tetrazolium (MTT) assay was used to evaluate the impact of USP39 on knockdown on cell viability. After 96 h of infection with recombinant lentiviruses, U2OS cells were washed and re-cultured in 96-well plates with 2000 cells per well. Each well was added adequate MTT solution and incubated for 4 h at 37 °C, and then added 100 µl acidic isopropanol (10% SDS, 5% isopropanol and 0.01 mol/L HCl) after the medium were carefully removed. The optical density was measured using microplate reader at the wavelength of 595 nm within 5 days. Triplicate repeats were carried out to determine the variance and significance.

### Colony formation assay

After 96 h of incubation with constructed recombinant lentiviruses, U2OS cells were washed and re-inoculated in the six-well plates at cell density of 600 cells per well, and incubated for 8 days to form natural monolayer colonies. Cells were washed and fixed by paraformaldehyde and washed twice with PBS solution, stained with crystals purple for 10 min, washed three times by ddH_2_O, and then photographed. The number of colonies (>50 cells/colony) was counted.

### Cell cycle and apoptosis analysis by flow cytometry

U2OS cells were inoculated into 6-cm dishes at a density of 2 × 10^5^ cells per dish for cell cycle test and 80,000 cells per dish for apoptosis after 40 h lentivirus infection. Propidium iodide (PI) and Annexin V/7-AAD were used to stain the cells following the manufacturer’s instructions. FAC Scan flow cytometer (Becton–Dickinson, San Jose, CA, USA) was then performed to measure the change of cell cycle and the percentage of apoptosis cells.

### Statistical analysis

All statistical analyses were performed using GraphPad Prism 5.0. The differences between groups were compared using Student’s *t* test, and data were expressed as mean ± SD of three independent experiments. Statistical difference was accepted at p < 0.05.

## Results

### USP39 is overexpressed in sarcoma

**Fig. 1:**
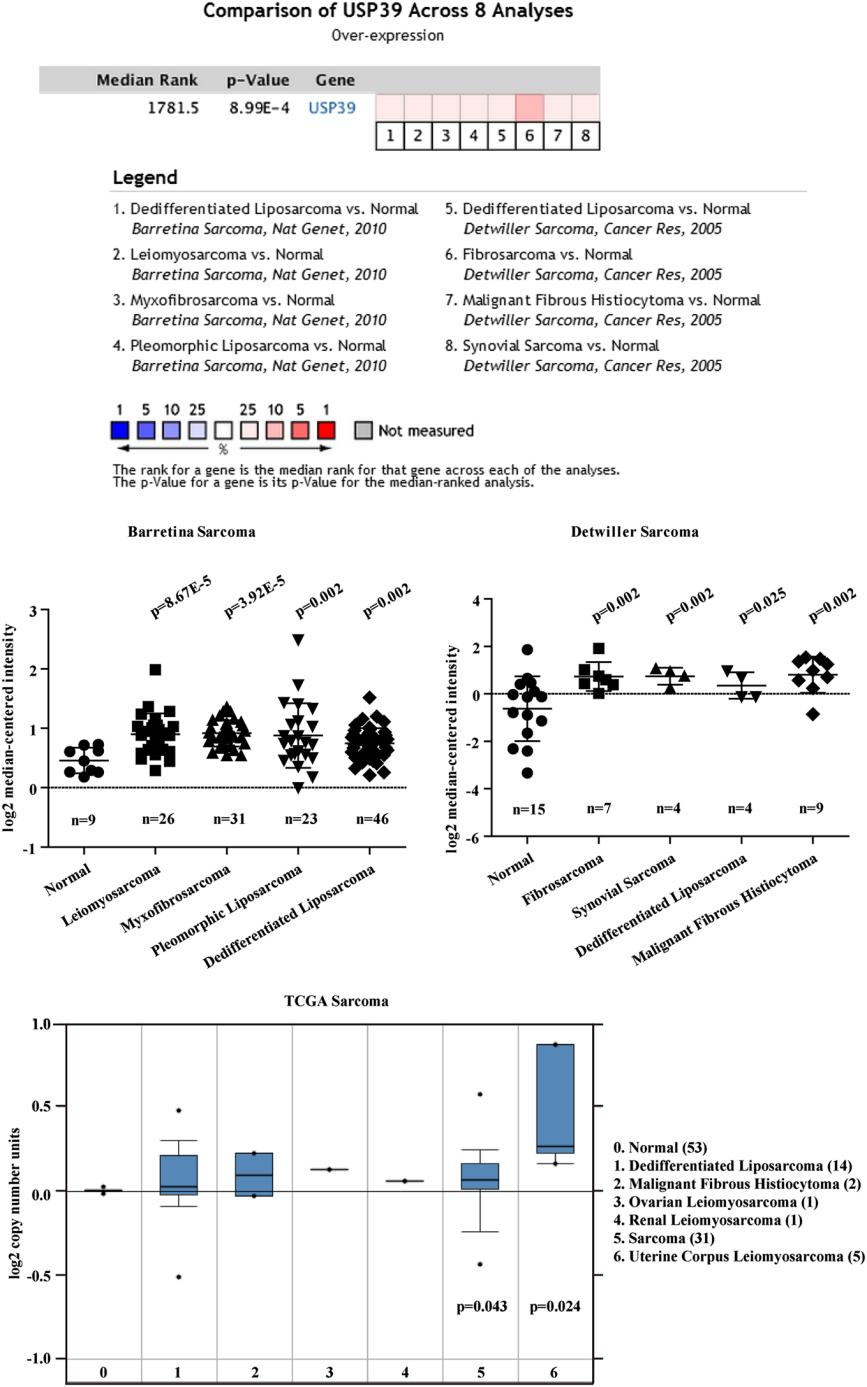
USP39 expression level in sarcoma tissues using the Oncomine database. Expression is shown as log2 transformation of median-centered intensity. *Black line* represents the median; *dots* represent minimum and maximum values. **a** mRNA level of USP in sarcoma tissues from Barrtina’s study. **b** mRNA level of USP39 in sarcoma tissues from Detwiller’s study. **c** The number of DNA copy of USP39 in sarcoma tissues from TCGA Sarcoma

To investigate the association of USP39 expression with sarcoma progression, Oncomine microarray data was used to evaluate USP39 gene expression in different sarcomas. The USP39 mRNA expression of all the sarcoma tissues in Barretina sarcoma and Detwiller sarcoma had a significant increase compared to that in normal issues (Fig. 1a, b, *p* < 0.05). Besides, DNA copies of USP39 in sarcoma tissues also have the same tendency as that of mRNA expression. (Fig. 1c, *p* < 0.05). Collectively, most of sarcoma tissues in Oncomine database had a higher expression of USP39 compared to that in normal issues.

### Lentivirus-mediated high-efficiency infection of U2OS cells for knockdown of USP39

**Fig. 2:**
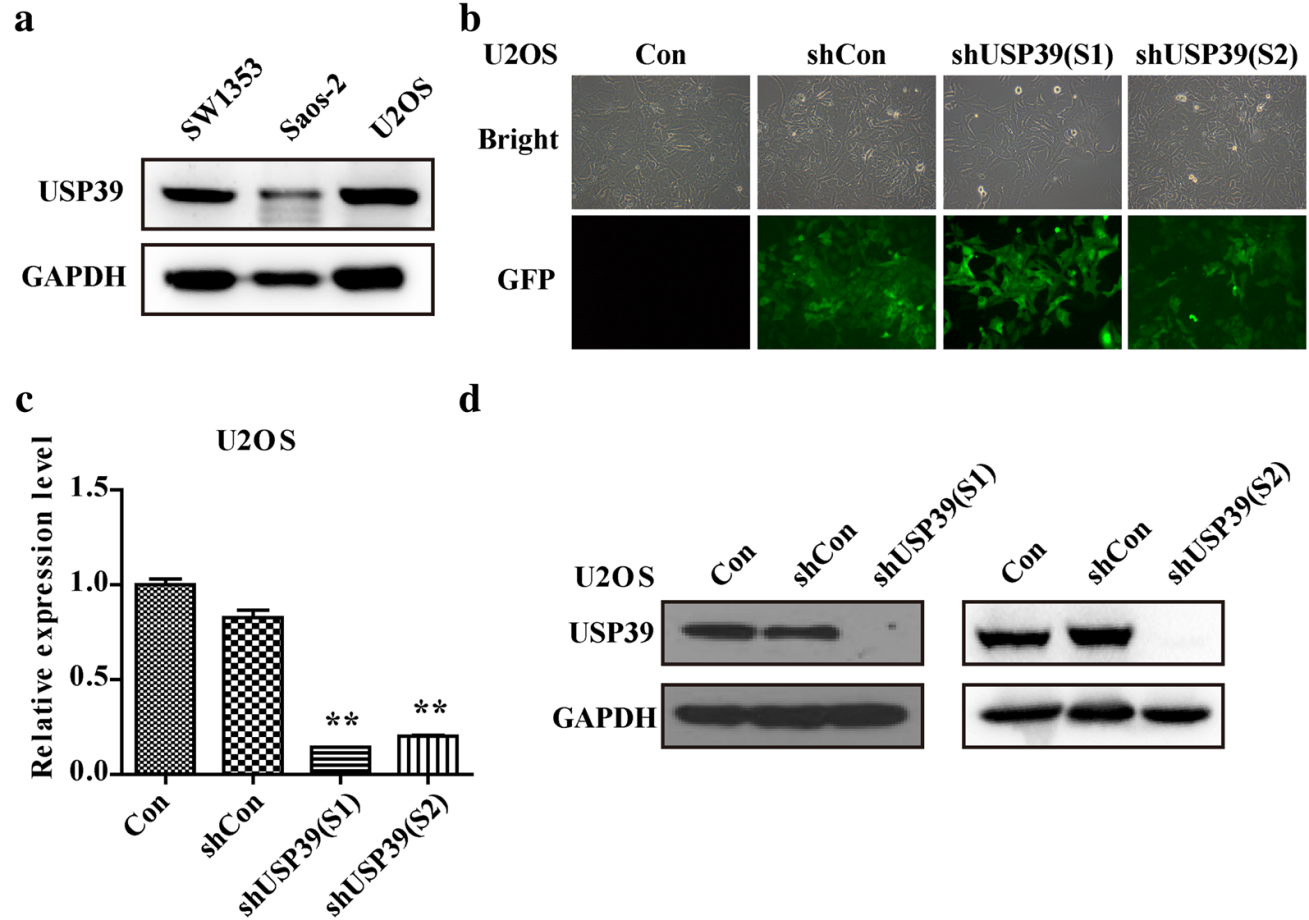
Knock down efficiency of USP39. **a** The expression level of USP 39 in different cell lines. **b** Microscopic images of osteosarcoma cells infected with lentivirus at MOI of 20 (magnification ×400). **c** Quantitative real-time RT-PCR was performed to monitor the knock down efficiency (Student’s *t* test, n = 3). **d** Western blot analysis of USP39 knockdown efficiency. **p < 0.01

We measured the expression level of USP39 in three different cell lines, including SW1353, SAOS-2 and U2OS. The results showed that U2OS had the highest expression level (Fig. 2a). Therefore, it was selected to study the effect of USP39 and knockdown of USP39 was conducted in U2OS cells. As shown in Fig. 2b, over 80% of osteosarcoma cells were GFP positive, indicating that the infection efficacy was satisfying. Subsequently, mRNA level and protein level of USP39 in U2OS after lentiviral infection were analyzed; the results were listed in Fig. 2c. USP39 mRNA expression was significantly down-regulated in U2OS after treatment with shUSP39 (S1) and hUSP39 (S2) respectively compared to that in shCon groups (p < 0.01). The protein expression level was also notably suppressed (Fig. 2d). Taken together, the lentiviruses constructed in this study have satisfactory efficiency.

### Knockdown of USP39 remarkably inhibited the cell proliferation of osteosarcoma cells

**Fig. 3:**
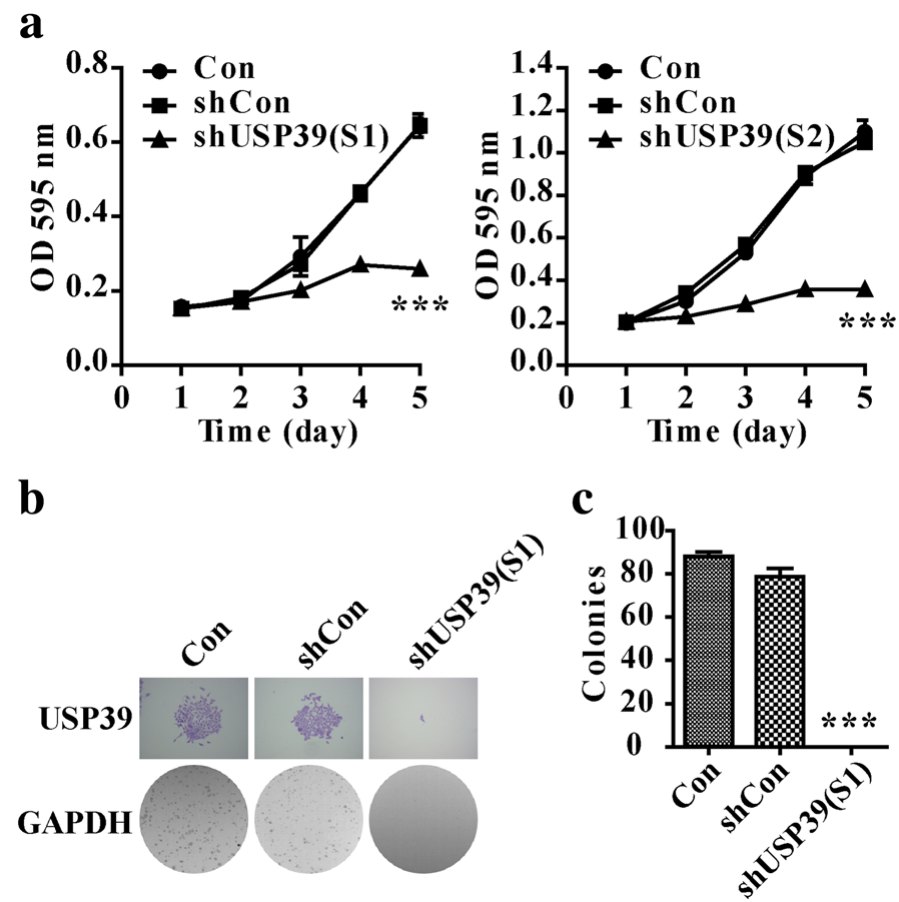
Down-regulation of USP39 inhibited U2OS proliferation ability and colony formation. After 96 h of infection with recombinant lentiviruses, U2OS cells were washed and re-cultured in 96-well plates with 2000 cells per well for MTT assay and in the six-well plates at cell density of 600 cells per well for colony formation. **a** Line chart of MTT assay was to determine the proliferation rate after shUSP39-S1 infection and shUSP39-S2 infection (Student’s *t* test, n = 3). **b** Representative microscopic images of colonies were stained by *crystal violet*. The full-sized vision of six-well plate under microscope showed significant colony formation inhibition in shUSP39 group. **c** Statistical analysis of the number of colonies (Student’s *t* test, n = 3). ***p < 0.001

To better understand the role of USP39 in osteosarcoma tumorigenesis, we examined the variation tendency of cell proliferation after lentivirus infection for 5 days. The growth of shUSP39 (S1) and shUSP39 (S2) infected U2OS cells was inhibited compared with shCon infected cells (Fig. 3a, p < 0.001),which indicated that knockdown of USP39 had an obvious effect on suppressing cell viability. Therefore, shUSP39 (S1) was used to evaluate the colony formation ability of U2OS cells after infection. The number of colonies decreased significantly in U2OS cells infected with shUSP39 (S1) compared with that in shCon group (Fig. 3b, c, p < 0.001). Collectively, knockdown of USP39 could markedly suppress the proliferation and colony formation ability of osteosarcoma cells.

### Knockdown of USP39 induced cell cycle arrest and its effect on cell cycle-regulatory molecules

**Fig. 4:**
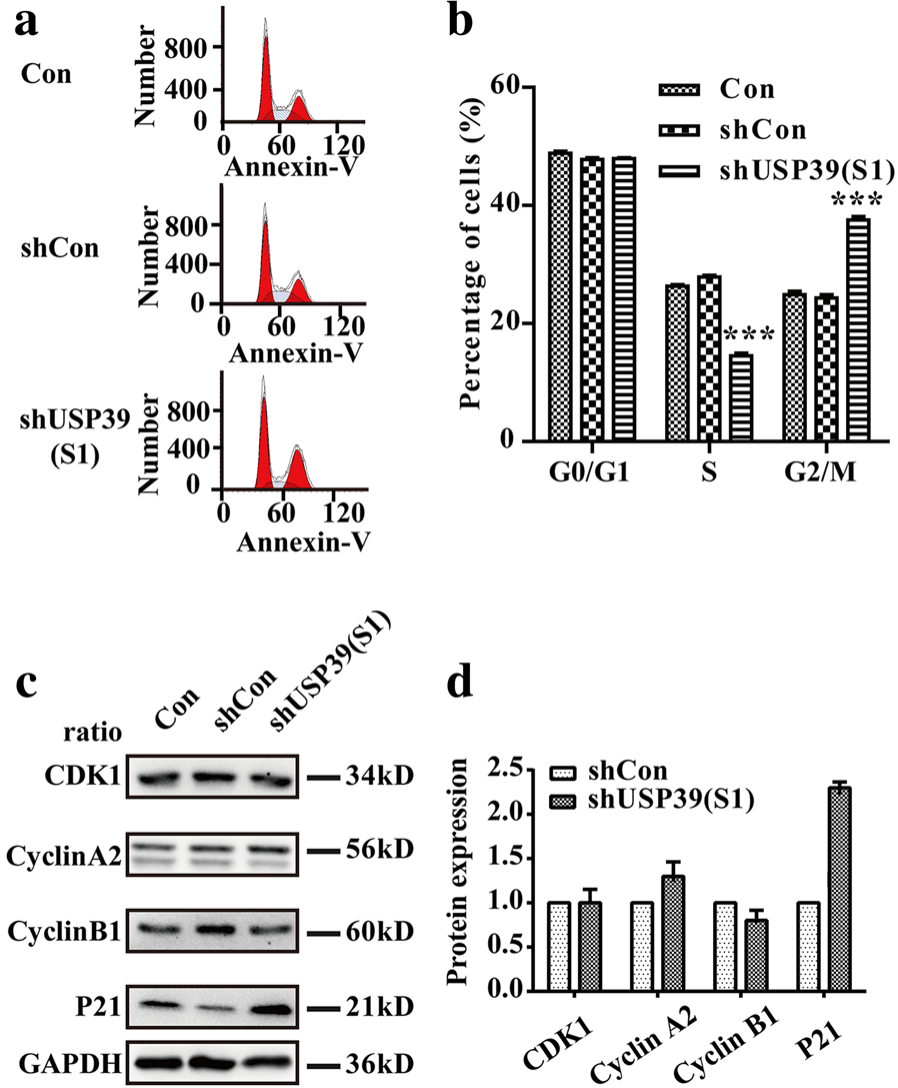
Depletion of USP39 induces G2/M phase cell cycle arrest in U2OS cells. U2OS cells were inoculated into 6-cm dishes at a density of 2 × 10^5^ cells per dish for cell cycle test and 80,000 cells per dish for apoptosis after 40 h lentivirus infection. **a** Cell cycle distribution of U2OS cells was analyzed by flow cytometry. **b** The population of cells in the G2/M phase was remarkably increased, accompanied by a noticeable reduction in the S phase (Student’s *t* test, n = 3). **c** USP39 silencing resulted in a cell cycle arrest via a cyclin-dependent way. **d** Statistic analysis of results in western blot (Student’s *t* test, n = 3). ***p < 0.001

To examine whether USP39 knockdown suppressed the growth of osteosarcoma cells through direct regulation of the cell cycle, the effect of USP39 repression on the cell cycle distribution was examined. Figure 4a indicated that cell distribution in cell cycles (G0/G1 phase, S phase and G2/M phase) varied significantly in three groups (Con, shCon and shUSP39-S1). As shown in Fig. 4b, the cell percentage of G2/M phase in shUSP39-S1 group (37.54%) was significantly increased, while the cell percentage of S phase (14.52%) was simultaneously decreased, by contrast to shCon group (S phase: 27.88%, G2/M phase: 24.34%), indicating that cell cycle was arrested in the G2/M phase. Our results demonstrated that treatment with shUSP39-S1 could remarkably induce cell cycle arrest (p < 0.001). These findings are in agreement with cell growth inhibition, which suggest that USP39 could modulate osteosarcoma cell growth via cell cycle control.

The mechanism by which USP39 knockdown arrested cell cycle might also affect the expression of negative and positive regulators of the cell cycle. To evaluate the role of cell cycle-regulatory molecules in knockdown of USP39-induced G2/M cell cycle arrest, we examined the effect of USP39 knockdown on cell cycle-regulatory molecules, including p21, CDK1 and cyclin A2 (Fig. 4c). There is no significance difference in the protein expression of CDK1, however, the protein expression of cyclin A2 and p21 significantly increased in the USP39 silencing U2OS cells. On the basis of these results, we hypothesized that knockdown of USP39 mediated cell-cycle arrest might occur through a cyclin-dependent manner in U2OS cells.

### Depletion of USP39 promotes apoptosis of U2OS cells

**Fig. 5:**
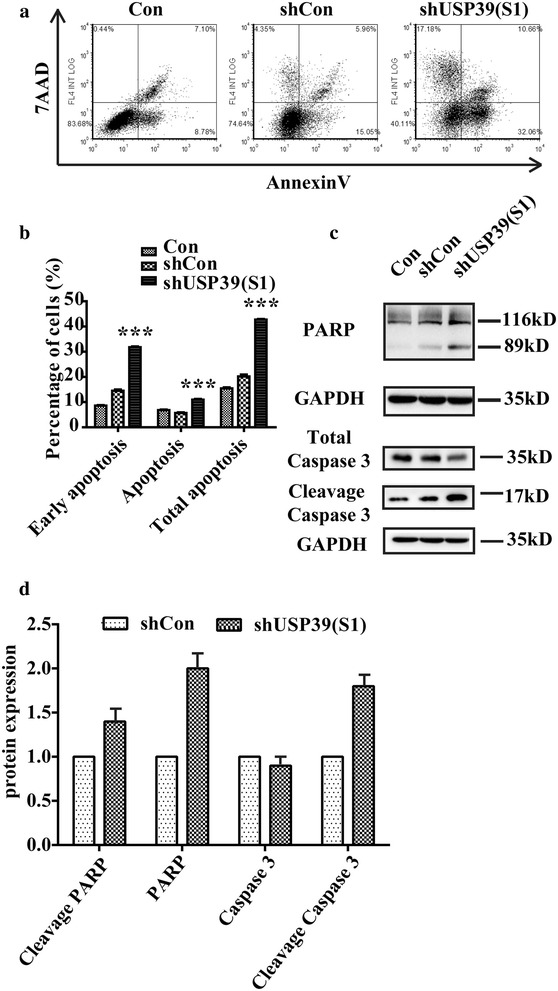
Depletion of USP39 induces apoptosis of U2OS cells. U2OS cells were inoculated into 6-cm dishes at a density of 2 × 10^5^ cells per dish for cell cycle test and 80,000 cells per dish for apoptosis after 40 h lentivirus infection. **a** Representative images showing Annexin V/7-ADD staining results. **b** Statistical data of Con U937 cells, shCon infected cells and Lv-shUSP39 infected cells. **c** The phosphorylation level of PARP was notably enhanced in the shUSP39 infected U2OS cells by western blot analysis (Student’s *t* test, n = 3). **d** Statistic analysis of results in western blot (Student’s *t* test, n = 3) ***p < 0.001

In order to demonstrate the effect of USP39 silencing on apoptosis of U2OS further, we performed flow cytometry using Annexin V and 7-aminoactinomycin D (7-AAD) as markers. As shown in Fig. 5a, b, about 31.77 ± 0.34% cells had early apoptotic properties (Annexin V +/7-AAD−) after USP39 knockdown, which was dramatically higher than Con group (8.62 ± 0.19%) and shCon infected group (14.51 ± 0.52%). The percentages of late stage apoptotic cells (Annexin V +/7-AAD+) are also higher after USP39 knockdown (10.98 ± 0.28% in shUSP39-S1 infected group compared with 6.65 ± 0.04% in Con group and 5.69 ± 0.23% in shCon infected group). These results suggested USP39 knockdown could promote U2OS cell apoptosis. Moreover, to uncover the mechanism governing the inhibitory effect of shUSP39 on cell growth, western blot analysis was utilized to detect the protein expression level of caspase 3 and PARP in the shUSP39 or shCon infected U2OS cells (Fig. 5c). A further analysis revealed that the cleavage caspase 3 and PARP were cleaved in the shUSP39 infected U2OS cells, indicating that the cleavage of PARP may be involved in shUSP39-mediated growth suppression in osteosarcoma cells.

## Discussion

In this study we identified and functionally characterized the effect of USP39 gene on human osteosarcoma. We demonstrated that down regulation of USP39 expression via RNA interference resulted in attenuated cell proliferation and colony formation. Meanwhile, knockdown of USP39 could arrest cell cycle at G2/M via cyclin-dependent pathway and promoted apoptosis through PARP cleavage [21–23].

Our research demonstrated that knockdown of USP39 had an effect on the cell cycle regulation, in which irreversible transition from one mitotic phase to the next by the complete deubiquitination of mitotic regulators plays an important role. As stated in the previous study, USP39 participated in the control of deubiquitination and splicing of aurora B [24] through encoding human tri-snRNP proteins [25]. Downregulation of aurora B, which was identified in humans as kinase providing functions in the attachment of the spindle to the centromere [26], affected the functional bipolar spindle by regulating the microtubule-depolymerizing activity of MCAK and Stathmin/Op18 [26]. Subsequent interrupted mitosis was observed when both of them were depleted [26]. Ubiquitin peptidase also affected p21 expression in breast cancer cell which inhibited the activity of cyclin-dependent kinase 1 (CDK1) [27], an essential regulator of mitosis as it controls the centrosome cycle as well as mitotic onset through forming a complex with cyclin A or cyclin B [28]. In our study, we have demonstrated that the expression of cyclin A2 and p21 increased which resulted in the arrest of cell cycle at G2/M phase after USP39 silencing, while the expression of CDK1 stayed the same. These results are consisted with previous study and demonstrated that USP 39 might affect cell cycle in cancer cell via a cyclin-dependent way.

It was reported that USP39-depleted cells failed to divide and retained a 4 N DNA content, and in a further study Van Leuken proved that the spindle checkpoint was defected when cells were treated with a vector-driven shRNA [11]. Therefore mitotic progress failed and apoptosis initiated. In our study we have supportive results demonstrating the population of U2OS cells was accumulated in quiescent G2/M phase. Moreover, USP39 silencing could promote U2OS cell apoptosis by PARP cleavage, which were reliable indicators of apoptosis.

In summary, our findings indicate that USP39 plays an important role in the cancer cell proliferation and cell cycle regulation in osteosarcoma, and depletion of it lead to retarded cell proliferation, triggered cell cycle arrest via p21-dependent way and promoted apoptosis through PARP cleavage. It was surprising to notice a complete blocking in cancer cell colony formation, which was indicating USP39 is critical for osteosarcoma tumorigenesis.

## Conclusion

The results uncover the critical role of USP39 in regulating cancer cell mitosis and indicate that USP39 is critical for osteosarcoma tumorigenesis.

## References

[1] Damron TA, Ward WG, Stewart A (2007). Osteosarcoma, chondrosarcoma, and Ewing’s sarcoma: national cancer data base report. Clin Orthop Relat Res.

[2] Dorfman HD, Czerniak B (1995). Bone cancers. Cancer.

[3] Unni KK (1998). Osteosarcoma of bone. J Orthop Sci.

[4] Ottaviani G, Jaffe N (2009). The epidemiology of osteosarcoma. Cancer Treat Res.

[5] Luetke A, Meyers PA, Lewis I, Juergens H (2014). Osteosarcoma treatment—where do we stand? A state of the art review. Cancer Treat Rev.

[6] Shimose S, Kubo T, Fujimori J, Furuta T, Ochi M (2014). A novel assessment method of serum alkaline phosphatase for the diagnosis of osteosarcoma in children and adolescents. J Orthop Sci.

[7] Valadkhan S (2007). The spliceosome: caught in a web of shifting interactions. Curr Opin Struct Biol.

[8] Lygerou Z, Christophides G, Séraphin B (1999). A novel genetic screen for snRNP assembly factors in yeast identifies a conserved protein, Sad1p, also required for pre-mRNA splicing. Mol Cell Biol.

[9] Makarova OV, Makarov EM, Lührmann R (2001). The 65 and 110 kDa SR-related proteins of the U4/U6·U5 tri-snRNP are essential for the assembly of mature spliceosomes. EMBO J.

[10] Liu S, Rauhut R, Vornlocher HP, Lührmann R (2006). The network of protein-protein interactions within the human U4/U6.U5 tri-snRNP. RNA.

[11] van Leuken RJ, Lunavargas MP, Sixma TK, Wolthuis RM, Medema RH (2008). Usp39 is essential for mitotic spindle checkpoint integrity and controls mRNA-levels of aurora B. Cell Cycle.

[12] Wang H, Ji X, Liu X, Yao R, Chi J, Liu S (2013). Lentivirus-mediated inhibition of USP39 suppresses the growth of breast cancer cells in vitro. Oncol Rep.

[13] Kim DH, Behlke MA, Rose SD, Chang MS, Choi S, Rossi JJ (2004). Synthetic dsRNA Dicer substrates enhance RNAi potency and efficacy. Nat Biotechnol.

[14] Guo W, Zhang Y, Chen T, Wang Y, Xue J, Zhang Y (2010). Efficacy of RNAi targeting of pyruvate kinase M2 combined with cisplatin in a lung cancer model. J Cancer Res Clin Oncol.

[15] Bischoff JR, Anderson L, Zhu Y, Mossie K, Ng L, Souza B (1998). A homologue of drosophila aurora kinase is oncogenic and amplified in human colorectal cancers. EMBO J.

[16] Sampath SC, Ohi R, Leismann O, Salic A, Pozniakovski A, Funabiki H (2004). The chromosomal passenger complex is required for chromatin-induced microtubule stabilization and spindle assembly. Cell.

[17] Kelly AE, Sampath SC, Maniar TA, Woo EM (2007). Chromosomal enrichment and activation of the aurora B pathway are coupled to spatially regulate spindle assembly. Dev Cell.

[18] Gadea BB, Ruderman JV (2006). Aurora B is required for mitotic chromatin-induced phosphorylation of Op18/Stathmin. Proc Natl Acad Sci USA.

[19] Rhodes DR, Yu J, Shanker K, Deshpande N, Varambally R, Ghosh D (2004). Oncomine: a cancer microarray database and integrated data-mining platform. Neoplasia.

[20] Livak KJ (2008). Analyzing real-time PCR data by the comparative C(T) method. Nat Protoc.

[21] Lazebnik YA, Kaufmann SH, Desnoyers S, Poirier GG, Earnshaw WC (1994). Cleavage of poly(ADP-ribose) polymerase by a proteinase with properties like ICE. Nature.

[22] Nicholson DW, Ali A, Thornberry NA, Vaillancourt JP, Ding CK, Gallant M (1995). Identification and inhibition of the ICE/CED-3 protease necessary for mammalian apoptosis. Nature.

[23] Cohen GM. Caspases: the executioners of apoptosis RID A-1687-2008. 1997. 10.1042/bj3260001 PMC1218630 9337844

[24] Zhang X, Lan W, Ems-Mcclung S, Stukenberg P, Walczak C (2007). Aurora B phosphorylates multiple sites on mitotic centromere-associated kinesin to spatially and temporally regulate its function. Mol Biol Cell.

[25] Elstein KH, Zucker RM (1994). Comparison of cellular and nuclear flow cytometric techniques for discriminating apoptotic subpopulations. Exp Cell Res.

[26] Riccardi C, Nicoletti I (2006). Analysis of apoptosis by propidium iodide staining and flow cytometry. Nat Protoc.

[27] Xiang T, Li L, Yin X, Yuan C, Cui T, Su X (2012). The ubiquitin peptidase UCHL1 induces G0/G1 cell cycle arrest and apoptosis through stabilizing p53 and is frequently silenced in breast cancer. Plos ONE.

[28] Malumbres M, Barbacid M (2009). Cell cycle, CDKs and cancer: a changing paradigm. Nat Rev Cancer.

